# Ultra-high density optical data storage in common transparent plastics

**DOI:** 10.1038/srep26163

**Published:** 2016-05-25

**Authors:** Deepak L. N. Kallepalli, Ali M. Alshehri, Daniela T. Marquez, Lukasz Andrzejewski, Juan C. Scaiano, Ravi Bhardwaj

**Affiliations:** 1 Department of Physics, Advanced Research Complex, 25 Templeton Street, University of Ottawa, Ottawa, K1N6N5, Ontario, Canada; 2 Department of Physics, King Khalid University, P.O. Box 9004, Abha, Saudi Arabia; 3 Department of Chemistry and Centre for Catalysis Research and Innovation, University of Ottawa, 10 Marie Curie, Ottawa, K1N 6N5, Ontario, Canada

## Abstract

The ever-increasing demand for high data storage capacity has spurred research on development of innovative technologies and new storage materials. Conventional GByte optical discs (DVDs and Bluray) can be transformed into ultrahigh capacity storage media by encoding multi-level and multiplexed information within the three dimensional volume of a recording medium. However, in most cases the recording medium had to be photosensitive requiring doping with photochromic molecules or nanoparticles in a multilayer stack or in the bulk material. Here, we show high-density data storage in commonly available plastics without any special material preparation. A pulsed laser was used to record data in micron-sized modified regions. Upon excitation by the read laser, each modified region emits fluorescence whose intensity represents 32 grey levels corresponding to 5 bits. We demonstrate up to 20 layers of embedded data. Adjusting the read laser power and detector sensitivity storage capacities up to 0.2 TBytes can be achieved in a standard 120 mm disc.

In conventional optical discs data storage is mostly confined to the surface of polycarbonate (PC). Data is stored in the form of pits and retrieved by a read laser whose reflection from the pits is detected by a photodiode. Disc capacity is determined by the bit density and depends on the wavelength of light and the numerical aperture (NA) of focusing optics used to record/read the data. Development of laser sources at shorter wavelengths and high NA optics resulted in smaller bit sizes and improved disc capacities. However, this planar technology cannot be scaled up due to the response and design of materials below 400 nm^1^. Storing information bits in a multi-layer structure using the three dimensional (3D) volume of the disc can overcome this limitation and provide ultra-high storage capacities due to the cubic dependence on the inverse of wavelength^2^,^3^.

3D optical data storage often relies on the nature of laser-induced changes in the recording medium in the form of refractive index/birefringence^2^,^3^, aggregation of metal nanoclusters^4^ or shape alteration of metal nanorods^4^,^5^. The technology relies on using ultrafast lasers to confine the changes in the medium on a micron scale^2^,^3^,^4^,^5^,^6^,^7^. Higher storage capacities could be achieved by overcoming the diffraction limit of light to record data on submicron scale and/or by multiplexing different properties of the light-matter interaction.

3D optical storage was first demonstrated by modifying the refractive index of photopolymers^8^ and glass^2^ locally on micron scale. Data was recorded *bit-by-bit* and retrieved by phase contrast microscopy. Recently, this technique was extended to demonstrate multilevel encoding of intensity and polarization states of light with self- assembled periodic nanostructures produced by an ultrafast laser in glass^2^,^9^. Data was retrieved by measuring the laser induced birefringence associated with the orientation of nanogratings. However, in both cases data retrieval is influenced by interference effects and/or low detection sensitivity limiting the number of data layers.

Data can alternatively be retrieved by fluorescence emission from the recorded bits. This has the added benefit of integrating the existing read technology. However, the recording medium had to be photosensitive. This led to a constant search for novel materials that are suitable for a recording medium^4^,^5^,^6^,^7^,^10^,^11^,^12^. Using silver doped zinc phosphate glass, 20 Gbits/cm^3^ of storage capacity was demonstrated by inducing embedded silver nanoclusters using an ultrafast laser^4^. Upon excitation with a read laser the nanoclusters emit fluorescence. Even higher capacities of up to 1 Tbits/cm^3^ were explored in 5D technology by utilizing the unique properties of surface plasmon resonance of gold nanorods stacked in multiple layers and using non-Gaussian laser beams to imprint the data^5^,^6^,^12^. Holography is an alternative technology capable of high-density data storage but it also requires photosensitive recording medium^13^,^14^,^15^.

In this article, we demonstrate high-density data storage in common inexpensive plastics. Clear plastics such as polymethylmethacrylate (PMMA), polycarbonate (PC), polydimethylsiloxane (PDMS), and polystyrene (PS) do not fluoresce in the visible spectrum. Irradiation by an ultrafast laser results in chemical changes followed by rearrangement leading to the formation of permanent defects^16^,^17^,^18^. The multiphoton nature of the laser-matter interaction confines these defects to very small volumes in the polymer matrix. These fluorescent moieties most likely derive from the polymer itself or from the additives that are used as stabilizers, such as UV protectors and antioxidants. Upon excitation with a read laser these defects emit fluorescence and the data is read in a confocal geometry. The fact that different excitation wavelengths lead to different emission profiles implies that more than one emitting chromophore is produced upon multiphoton excitation. The simplicity of our approach enables data access with multiple excitation sources unlike other techniques. The stored data can be embedded in the bulk material and is thermally stable up to the glass transition temperature of the recording medium thereby offering a long shelf life. Our technique eliminates the need for the recording medium to be photosensitive and the multiple steps involved in their preparation.

## Results

### Fluorescence from femtosecond laser recorded data bits – multi-level encoding dynamics

**Figure 1:**
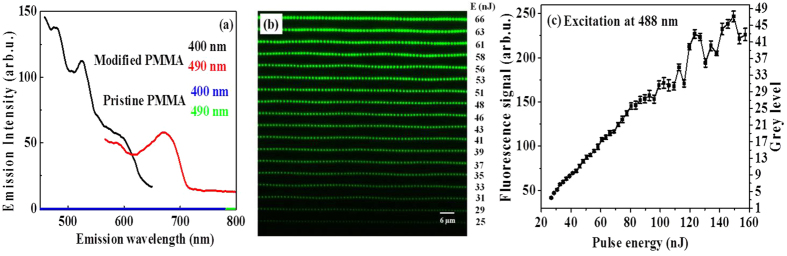
Fluorescence in PMMA from laser irradiated regions. (**a**) Fluorescence (emission) spectra from pristine and modified PMMA at 400, and 490 nm excitations obtained with a conventional fluorescence spectrometer. A large area grating like structure was fabricated with 300 *μ*m inside PMMA. Only laser irradiated regions fluoresce. (**b**) Confocal fluorescence microscope image of ultra-fast laser modified regions. Excitation wavelength was 488 nm and emission was recorded in the wavelength range of window 500–550 nm. Each row shows fluorescence from a periodic array of modified regions irradiated with a single laser pulse of specific energy. Fluorescence signal increased with pulse energy from bottom to the top row as shown on the right. (**c**) Evolution of fluorescence signal with the energy of a single fs pulse obtained from (**b**). Based on the fluctuation of the fluorescence signal grey level was assigned to fluorescence as shown on the right ordinate.

Figure 1a shows the fluorescence emission spectrum in PMMA for pristine (blue and green curves) and laser modified regions (black and red curves in Fig. 1a) at two different excitation wavelengths. Pristine PMMA does not fluoresce in the visible spectrum. The fluorescence moieties induced by the femtosecond laser exhibit different emission profiles upon excitation at different wavelengths. The fluorescence signal intensity from the ultrafast laser irradiated regions within the material varied with the pulse energy used to induce the emissive centres. Figure 1b shows confocal fluorescence microscope image obtained with 488 nm excitation. Each row consisted of a series of laser-modified regions irradiated by a single laser pulse at a specific energy with a pitch of 1.4 *μ*m and successive rows were separated by 5 *μ*m. The minimum energy required to induce fluorescence in PMMA with a single laser pulse was ~25 nJ below which there was no detectable fluorescence in the emission window of 500–550 nm. The fluorescence intensity increased with the pulse energy of the ultrafast laser up to ~130 nJ above which the modified regions started to overlap. Further increase in the pulse energy resulted in formation of voids with no increase in the fluorescence intensity.

The evolution of fluorescence signal with the energy of the recording laser was used to assign different grey levels. This unique feature enables to represent each modified region with several bits of data, increasing the storage capacity. Figure 1c shows the division of the fluorescence signal into different grey levels associated with different pulse energies of the recording laser. The absence of fluorescence signal below 25 nJ represented level 0. The linearity of the fluorescence signal with pulse energy enabled us to assign 32 levels corresponding to 5 bits of data. Similar results were obtained with a different read/excitation laser (see Supplementary Figure S1) and in different materials PDMS, PS and PC (see Supplementary Figure S2). This flexibility offers the data to be stored in commonly available plastics and be accessed at any excitation within the visible spectrum.

### Recording and retrieving 5-bit images in 3D

**Figure 2:**
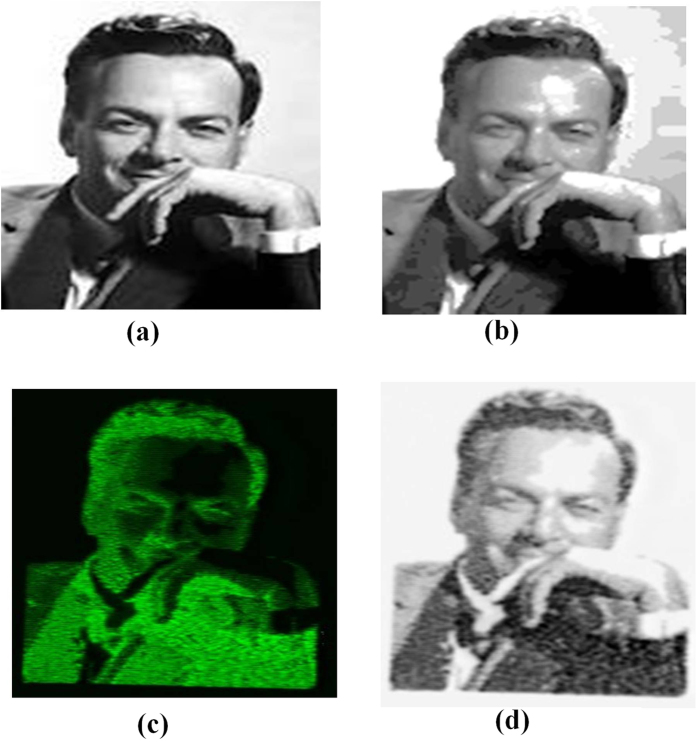
5-bit (32 grey-level) image fabrication. (**a**) Multi-grey image of Richard Feynman - Nobel laureate in physics in 1965 (105 pixels × 147 pixels). (**b**) Processed image after assigning 32 grey levels corresponding to write laser pulse energy range of 25–130 nJ. (**c**) Confocal fluorescence microscope image of (**b**) obtained by a CW, 488 nm read laser at 100 *μ*W of power. (**d**) Reconstructed negative image of (**c)**. This figure is not covered by the CC BY license. [Credits to Mary Evans Picture Library/The Canadian Press]. All rights reserved, used with permission.

The usefulness of inexpensive plastics for data storage applications is demonstrated in Fig. 2 where a black and white 5-bit image (32 grey levels) of Richard Feynman was recorded inside PMMA with an ultrafast laser (Fig. 2a,b). Using a 488 nm CW laser in conjunction with a confocal microscope the fluorescence from the recorded image was retrieved and decoded (Fig. 2c,d). The size of the original image was 105 × 147 pixels (Fig. 2a). Assigning the 32 grey levels to the pulse energies in the range of 25–130 nJ resulted in the processed image, Fig. 2b, which was used to record the image. The pixel spacing of the recorded image was 1.4 *μ*m with a pixel size that varied with pulse energy up to a maximum of 1.1 *μ*m (see Supplementary Figure S3). The pixel spacing was chosen to minimize the cross talk between two adjacent pixels representing the highest grey level. High laser pulse energies are required to record such pixels resulting in large pixel size. Fluorescence from each pixel, when excited by the read laser, represents a particular grey level (Fig. 2c). A negative of the retrieved fluorescence image reproduced the recorded data (Fig. 2d).

**Figure 3:**
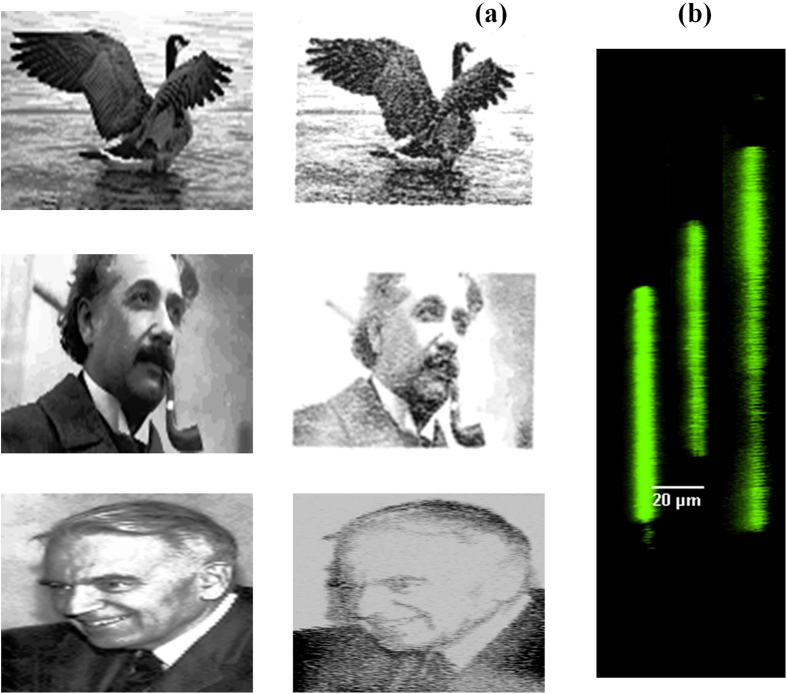
3D stacked 5-bit images. (**a**) Set of three 32 grey level images of a Canada goose (113 pixels × 75 pixels), Albert Einstein -Nobel laureate in Physics in 1921 (145 pixels × 87 pixels), and Gerhard Herzberg - Nobel laureate in Chemistry in 1971 (81 pixels × 113 pixels) stacked on top of each other with a spacing of 20 *μ*m. The left column shows the individual processed images and the right one shows the images recovered by confocal fluorescence microscopy at an excitation wavelength of 488 nm. (**b**) Lateral view of 3D stack of confocal fluorescence microscopy images shown in (**a**). Image of Canada goose is covered by the CC BY licence.[Credits to the Nature mapping foundation for usage of this image http://naturemappingfoundation.org/natmap/facts/canada_goose_k6.html, http://www.naturespicsonline.com/, License to use the image is released under http://creativecommons.org/licenses/by-sa/3.0/]. The image for Albert Einstein is not covered by CC BY license. [Credits to Getty Images for Albert Einstein]. All rights reserved, used with permission. The image of Gerhard Herzberg is not covered by CCBY license. [Credits to Mary Evans Picture Library/The Canadian Press]. All rights reserved, used with permission.

The 3D data storage capability of our technique is demonstrated in Fig. 3a where a stack of three images of Canada goose, Albert Einstein and Gerhard Herzberg, respectively, were embedded 200 *μ*m below the surface of PMMA with a 20 *μ*m layer separation. In all cases the original images were downsized to reduce the fabrication time and were assigned grey levels (left column). The decoded images, obtained by optical sectioning of the modified region and stacking the fluorescence signal, were shown in the right column. A 40 *μ*m^3^ volume (with a pixel spacing 1.4 *μ*m, 20 *μ*m layer separation) represents 5 bits of data leading to a maximum storage capacity of 127 Gbits/cm^3^. Practical storage capacity that can be achieved will be lower as discussed below. The lateral view of the 3D stack of Fig. 3b shows no crosstalk between the layers. Each fluorescent bit had a depth of nearly 8 *μ*m due to confocal parameter of focusing optics (0.9 NA). It is therefore possible to reduce the layer spacing further to 10 *μ*m and subsequently increase the maximum storage capacity to 0.25 Tbits/cm^3^ (see Supplementary Figure S4).

**Figure 4:**
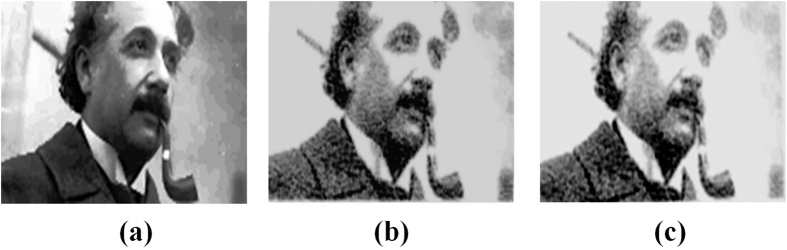
Faster image retrieval – 3D stack vs single section. Decoded 32-grey level images of Nobel laureate Albert Einstein after 3D-stacking all the sections (middle panel), and from the single intense plane of the stack (right panel). Image on left was used to fabricate inside PMMA. Recorded images were read using confocal fluorescence microscope at 488 nm excitation (500–550 nm emission). This figure is not covered by the CC BY licence. [Credits to Getty Images]. All rights reserved, used with permission.

Optical sectioning and stacking can be time consuming but is not critical. A single intense central plane can be sufficient to retrieve the recorded images without loss of information and therefore reduce the read time. Figure 4 shows 5-bit images of Einstein; (a) was used to record the image, (b) was retrieved by stacking different layers while (c) was obtained from a single plane.

**Figure 5:**
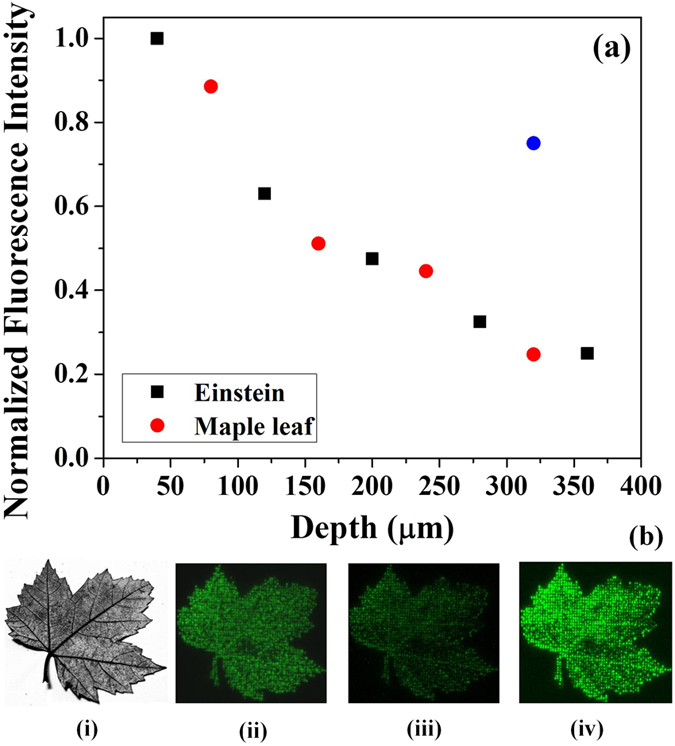
(**a**) Variation of integrated fluorescence signal with the number of stacked images. The integrated fluorescence signal from the embedded layers of 3-bit images of Albert Einstein (black squares) and a maple leaf (red circles) decreased with the number of layers. The layer spacing was 40 *μ*m. Read laser wavelength was 488 nm and the power was 100 *μ*W. The blue data point corresponds to a read laser power of 340 *μ*W. (**b**)Fluorescence optimization i. Grey image (8 levels) of maple leaf used for fabrication at different depths in PMMA. ii, iii. Fluorescence images of maple leaf at 200, and 360 *μ*m below the surface, respectively, obtained using 488 nm light at 100 *μ*W of power, and the detector sensitivity of 82 V. iv The reduced fluorescence signal of image (iii) at a depth of 360 *μ*m could be recovered by increasing the read laser power to 340 *μ*W. The image of maple leaf is not covered by the CC BY licence [Credits to Marc P. Bergen. https://rightinfrontofme.wordpress.com/tag/maple-leaf/]. All rights reserved, used with permission.

The multiphoton nature of the interaction of ultrafast lasers with transparent dielectrics is known to enable recording multiple layers of data. However, retrieving the data can be affected by the transmission of the read laser through different layers of the modified material. Figure 5a shows the variation of the integrated fluorescence signal obtained from individual images embedded at different depths in PMMA, which were normalized to the image closest to the surface. 3-bit images (8-grey levels) of Albert Einstein and a maple leaf were fabricated at different depths alternately, and were separated by 40 *μ*m. The images were retrieved using confocal microscope with 488 nm read laser at the same power and detector sensitivity. The reduction in the normalized fluorescence signal can be attributed to losses in transmission of the read laser and scattering of the fluorescence from layers above.

In optical data storage technology, the number of layers of data that can be retrieved without loss of information is one of the factors that plays a major role in disc capacity. The reduction of fluorescence signal observed in Fig. 5a can lead to loss of information but could be easily recovered either by increasing the detector sensitivity and/or the read laser power. Figure 5b(i) shows the image of maple leaf (8-grey levels) used for recording at different depths. For a fixed power of the read laser (100 *μ*W of 488 nm light) and the detector sensitivity (82 V), Fig. 5b(ii,iii) show the retrieved fluorescence images at 200 *μ*m and 360 *μ*m below the surface, respectively. The decrease in fluorescence signal for the maple leaf at 360 *μ*m (Fig. 5b(iii)) was due to light scattering from the seven embedded images above it. Upon increasing the read laser power to 360 *μ*W, the fluorescence signal could be recovered as shown in Fig. 5b(iv) with a three fold increase in intensity (Fig. 5a).

### Photo-recovery and thermal stability of recorded images

**Figure 6:**
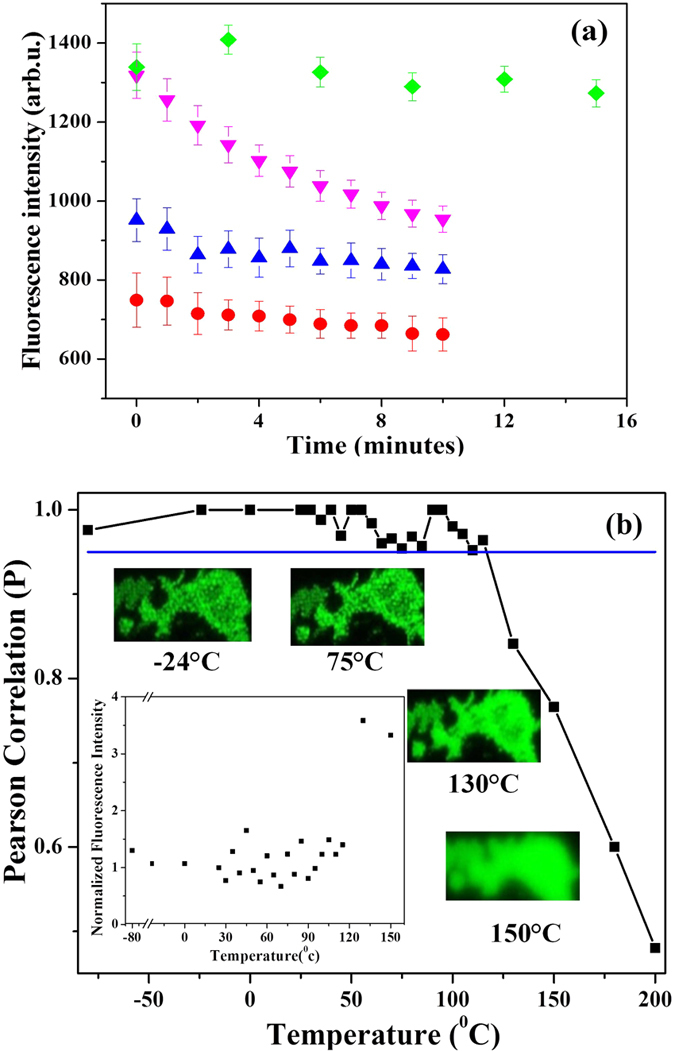
Photo-recovery and thermal stability of embedded data. (**a**) Recovery of fluorescence signal from fs laser-modified polymer using different *on-off* duty cycles of the 488 nm read laser at different powers and detector’s sensitivity. The *on* time was fixed at 30 s. For fixed *off* time of 1 minute and a sensitivity of 100 V the fluorescence signal was high at 200 *μ*W of read laser power (blue up triangles) compared to 100 *μ*W (red circles) and no degradation with time was observed. However, at high power (340 *μ*W power and sensitivity of 90 V) the fluorescence signal does not recover sufficiently (pink down triangles). The recovery is better when the *off* time was increased to 3 minutes (green diamonds). (**b**) Plot of Pearson correlation of images of random pattern at different temperatures relative to the image at room temperature (25 °C). Inset shows the variation of integrated fluorescence signal at different temperatures normalized to the image obtained at room temperature.

Higher powers of the read laser raise concerns of photo-recovery of the polymer during multiple read cycles. Recovery of the fluorescence signal from the defects induced by the write laser for different *on-off* duty cycles of the read laser is shown in Fig. 6a. All measurements were made on the top image layer with different incident powers. In contrast, in Fig. 5 the power delivered to images at different depths is not the same as the incident power due to losses. In all cases the read laser was *on* for 30 seconds. At low powers (100–200 *μ*W) there is no appreciable change in the fluorescence signal when the read laser was *off* for a minute. At a higher power of ~340 *μ*W and an *off*-time of 1 minute there is a decay of the fluorescence signal with multiple read cycles. Increasing the *off* time to 3 min improved the photo-recovery. The florescence signal did not diminish when the data were read after several days and weeks.

Environmental effects influence the long-term stability and shelf life of any data storage system. Specifically, thermal stability is crucial for practical implementation^19^,^20^,^21^,^22^. Polymer properties are known to thermally degrade and therefore can alter the fluorescence emitted by the ultrafast laser irradiated regions. Figure 6b shows the correlation coefficient of fluorescence images obtained at different temperatures with respect to that at room temperature. In the temperature range of −80 °C to 115 °C, the correlation coefficient is close to unity suggesting the images are spatially correlated with similar pixel intensities. Also, shown are fluorescence images at different temperatures. Beyond 130 °C the image quality degraded with poor spatial resolution although the fluorescence intensity increased significantly (inset of Fig. 5b). In the temperature range of −80 °C to 115 °C the normalized fluorescence signal, shown in the inset of Fig. 6b, does not vary significantly suggesting the stored data is stable up to the glass transition temperature of PMMA^23^. At temperatures beyond 200 °C, close to the melting point, the images are blurred as the modified regions coalesce together. However, the fluorescence does not disappear. So, unlike in some glasses the stored data cannot be erased and rewritten by raising the temperature to glass transition.

## Discussion

**Figure 7:**
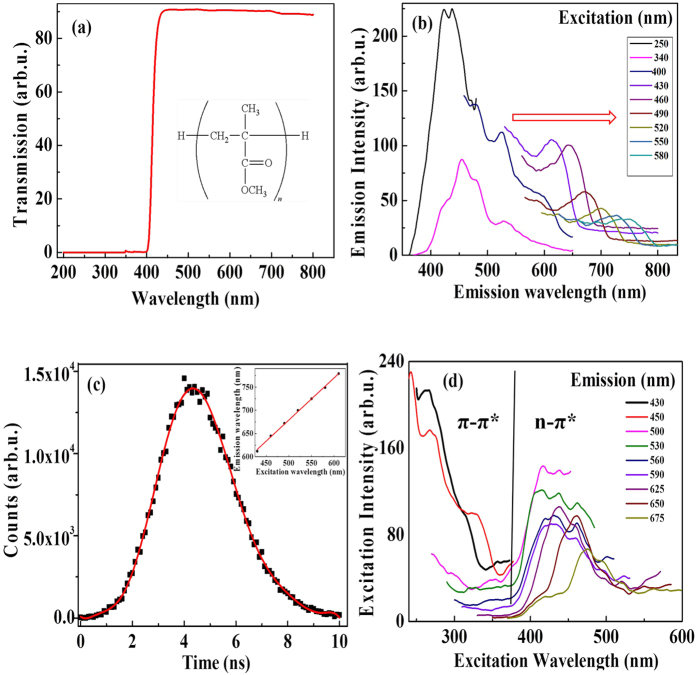
Spectral analysis. (**a**) Transmission spectrum of pristine PMMA (chemical structure shown in inset). (**b**) Fluorescence (emission) spectra recorded for ultra-fast laser modified PMMA at different excitations covering the entire visible spectrum. Red shift in fluorescence peak with excitation wavelength is indicated by an arrow. (**c**) Fluorescence lifetime measurement from the ultrafast-laser modified region. Inset shows the linear red shift of the fluorescence peak (as indicated in **b**) observed from 400 nm onwards with different excitation wavelengths (**d**). Excitation spectra of the laser modified PMMA recorded at different fluorescence emission windows. Regions of possible (*n* − π*), and (*π* − π*) transitions are indicated in the figure, demarcated by a line around 380 nm.

To comprehend the nature of laser-induced fluorescence and its origin we recorded the transmission, emission and excitation spectra using conventional transmission and fluorescence spectroscopy. The commercially obtained pristine PMMA sample was tested for its spectral purity using a Cary 100 UV-vis absorption spectrometer. Figure 7a shows the UV-visible absorption spectrum of pristine PMMA (chemical structure is shown in inset of Fig. 7a) recorded in transmission mode. No light is transmitted below 400 nm (3 eV photon energy) suggesting additives have been added to the polymer to provide UV protection (note: the bangap of pure PMMA is 4.58 eV^24^,^25^). The embedded UV absorbers/stabilizers, although not disclosed by the manufacturer, typically contain aromatic moieties, carbonyl groups and phenols (specifically, quinone and benzophenone families as well as propionate and benzotriazole derivatives^26^,^27^,^28^,^29^). Upon 2-photon excitation these groups lend themselves to photochemical transformations leading to fluorescent molecules that emit light upon excitation. Very low transmittance below 400 nm, as observed in the spectrum of unmodified PMMA samples, indicates the potential use of more than one type of UV absorbers.

Pristine PMMA does not exhibit fluorescence in the visible spectral range (Fig. 1a), while laser-modified PMMA does emit fluorescence. Figure 7b shows fluorescence emission spectra from laser modified PMMA. Fluorescence emission could be attributed to either the formation of nano-clusters of different sizes^30^ or rearrangement of different aromatic moieties upon two-photon absorption that leads to the formation of fluorophores confined in a rigid polymer matrix. The fluorescence intensity not only decreased with increasing excitation wavelength but also shifted to the red-edge of the excitation spectrum.

The previous assumption of the existence of more than one type of stabilizers can be supported by the broad lifetime distribution obtained by Fluorescent Lifetime Imaging Microscope (FLIM) as shown in Fig. 7c. One would normally expect that emission from a single compound be independent of the excitation wavelength, following Kasha’s rule^31^,^32^. Here the marked dependence of emission on the excitation wavelength (red shift shown in inset of Fig. 7c) is a clear indicator that multiple (single) chromophores (of varying sizes) are involved likely due to the use of UV stabilizers and the formation of diverse emissive products upon high intensity laser excitation. The measured lifetime distribution was centered at 4.2 ns with a FWHM of 3.4 ns, too broad to be attributed to a single emitter. The evidence that laser excitation causes similar effects independently of the diverse nature of the polymers is consistent with the photo-degradation of the UV stabilizers, as these molecules are widely employed in the polymer industry. The broad lifetime of the fluorescence signal obtained using FLIM technique also rules out the possibility of Raman scattering.

Figure 7d shows the excitation spectra recorded for laser modified PMMA. From the fluorescence spectra obtained in Fig. 2b, we recorded the excitation (absorption) spectrum for each emission. First, two distinct excitation bands could be observed below and above approximately 380 nm. The origin of these bands could be attributed to (n − π*) and (*π* − π*) transitions of fluorophores in aromatic molecules^18^,^30^,^33^,^34^. In literature, maximum excitation around 400 nm was ascribed to the absorption of some unsaturated aldehyde or ketone groups, that undergo (n − π*) transition^18^,^33^,^34^,^35^. The energy associated with (n − π*) transition is lower compared to (*π* − π*) transition that typically corresponds to UV absorption below 380 nm^34^. Second, the (n − π*) bands were found to be shifting with emission from 380 nm onwards as shown in the inset of Fig. 7d. This feature enables us to use a broad range of read laser wavelengths to retrieve the recorded data. To conclude, laser treated plastics result in the formation of aromatic compounds (fluorophores) that undergo (n − π*) and (*π* − π*) transitions upon suitable excitation.

Standard optical discs (CD and DVD) are made of 1.2 mm thick polycarbonate with a recording area of ~100 cm^2^. With a 1.4 *μ*m pixel spacing, a single layer of data in the disc holds 25 Gbits with 5-bit encoding. When data is embedded 150 *μ*m below the surfaces the effective thickness available for recording is 900 *μ*m. By tailoring the read laser power and detector sensitivity the number of layers that can be imaged without loss of information can be extended up to 30. Reading the data on both sides of the disc allows one to stack 60 layers with a spacing of 15 *μ*m (each fluorescent bit had a depth of 8 *μ*m due to confocal parameter, Fig. 3d) providing a storage capacity of 0.2 TBytes/disc. Using high NA microscope objective, pixel spacing can be reduced further to 1 *μ*m and with a layer spacing of 10 *μ*m data storage of 0.5 TBytes/disc is feasible.

Our data storage technique has key advantages. (1) The material need not contain photosensitive molecules to start with – the write laser induces them. (2) Cost effectiveness – commonly available plastics can be used. (3) No special sample preparatory steps are required compared to other techniques and large discs can be readily available. (4) Adaptable with existing DVD and Bluray technology. The power of the read/excitation laser (both 405 and 488 nm) used in our measurements is in the range of 100 *μ*W–1 mW comparable to DVD/Bluray. So, the read laser requirements are minimal and only the detection mechanism needs to be modified to the confocal configuration. (5) Possesses unique capability of data retrieval with any excitation source in the visible spectrum. (6) The storage discs have long shelf life with operable temperatures in the range of −80 °C to 115 °C. Tailoring the glass transition temperature of the polymers by general doping or controlling the residual solvent in polymer can further extend this range. The main drawback of our technique is that the discs are read only.

## Methods

### Materials

The commercially available PMMA sample (CQ grade, Goodfellow, UK) showed strong absorbance throughout the ultraviolet region (see Fig. 7a) and had 50% transmittance at ~415 nm. Thus, the additives that provide excellent UV protection to these polymers are also suitable for two-photon absorption at 800 nm. While the UV absorbers utilized by the manufacturer are not disclosed, common additives contain aromatic moieties, carbonyl groups and frequently phenols, all structures that lend themselves to photochemical transformations leading to fluorescent molecules as observed in this contribution.

### Micro-fabrication of data

800 nm light from a Ti: Sapphire laser amplifier system operating at a repetition rate of 1 KHz and producing 45 fs pulses with maximum pulse energy of 2.5 mJ was used to record data in PMMA. Laser pulses were focused at desired depth below the surface of PMMA by a high numerical aperture (NA = 0.9) microscope objective. The back aperture of the microscope objective was slightly over filled to minimize alignment errors. The position of the laser focus relative to the surface of bulk PMMA sample was accurately determined by imaging the back-reflected light with a CCD camera at very low pulse energies (5 nJ) below the ablation threshold. The PMMA samples (2.5 cm × 1.3 cm surface area with 3 mm thickness) were cleaned with methanol and were mounted on three-axis translation stages (Newport) with a bidirectional repeatability of 100 nm along the lateral dimensions (X, Y) and 200 nm along the axial direction (Z). A combination of a half-wave plate and polarizer were used to vary the pulse energy. The incident pulse energies were measured before the microscope objective. A single-shot auto-correlator continuously monitored the pulse duration. A single laser pulse was used to record a data bit.

### Multi-level encoding dynamics

An array of periodic laser-modified regions were fabricated to study multi-level encoding dynamics. Each row corresponded to a specific energy and consisted of a set of 140 modified regions. Pulse energy was varied from 10 nJ to 920 nJ in small increments. A water immersion microscope objective (Olympus, LUMPlan F1/IR, 0.9 NA, 60X magnification, 2 mm working distance) was used to focus light 100 *μ*m below the PMMA surface. The spacing between each modified region in a row was 1.4 *μ*m obtained by scanning the sample at a speed of 1.4 mm/s. Each successive rows (corresponding to different pulse energies) were separated by 5 *μ*m. Fluorescence from the laser-modified regions was recorded using Nikon A1RMPST confocal fluorescence microscope with a 25X objective (NA 1.1, working distance 2 mm, water immersion) at two excitation wavelengths of 488 nm and 405 nm with emission windows in 500–550 nm and 425–475 nm range, respectively. The power of the CW read laser and sensitivity of the detector for 488 nm (405 nm) excitation were 100 *μ*W and 82 V (200 *μ*W and 142 V), respectively. Each fluorescence image recorded 18 rows of modified regions corresponding to different pulse energies. Successive images consisted of 3 common rows of modified regions to normalize the fluorescence intensity. Image J software was used to evaluate mean and standard deviation of fluorescence by averaging 15–20 modified regions for each energy. The fluctuation in the fluorescence intensity was then used to divide the linear dependence of the fluorescence signal on ultrafast laser pulse energy into multiple grey levels as shown in Fig. 1c. 5-bit images were also analyzed by Image J to obtain 3D stacks. Each reconstructed image consisted of 20 layers.

### Image fabrication

The raw image was first spatially filtered to remove any artifacts. Pixels of similar value were then clustered before the image was re-sampled to a printable resolution. Following histogram equalization, the image was subdivided into the desired number of grey levels as determined by its intensity distribution. To speed up the printing process and minimize wear on the translation stages, pixels of identical value were grouped into particles for which an individual map of coordinates were generated. Once a focal plane was determined, printing of an image was achieved by scaling the energy range of the laser to the corresponding grey level and by sending the scaled pixel coordinates of particles, one by one, to the motion stage controller, while triggering the laser for each pixel.

### Spectral analysis

Fluorescence from small volume of the laser-modified region (~10 *μ*m^3^) is difficult. We therefore fabricated a grating like structure in PMMA 300 *μ*m below the surface in an area of 2 mm × 4 mm with a pitch of 2 *μ*m at scan speed of 0.2 mm/s with an energy of 200 nJ using 0.25 NA microscope objective (16X magnification). A conventional fluorescence spectrometer (PerkinElmer LS 50) with a slit width of 15 nm and spectral resolution of 1 nm was used to record both the emission and excitation spectra. Water Raman signals and a reference fluorescent dye were used to calibrate the spectrometer. Fluorescence spectra were recorded for modified PMMA in the range of 250–610 nm with increments of 30 nm.

### Packing density

The number of images that can be stacked without loss of information during the retrieval process determines the packing density and was obtained by measuring the variation of the normalized fluorescence signal with depth. 3-bit embedded images of Albert Einstein (80 pixels × 103 pixels) and a maple leaf (100 pixels × 100 pixels) were fabricated at different depths alternately separated by 40 *μ*m, using 0.9 NA, 60X microscope objective with laser pulse energy range 25–130 nJ. Confocal fluorescence microscope images were recorded with 488 nm excitation (power 100 *μ*W, sensitivity 82 V). An integrated fluorescence signal from each image was obtained using Image J and normalized to the image fabricated closest to the surface.

### Photo-recovery of the fluorescence signal

Prolonged exposure of laser modified region to the excitation laser was studied by repeatedly turning on and off the laser with a specific duty cycle. The duty cycle consisted of an *on* time of 30 s to record fluorescence from the 3D stack followed by an *off* time of either 1 or 3 minutes. The variation of fluorescence signal recorded during each *on* cycle enabled us to study photo-recovery of the modified polymer. For this study, an array of periodic laser-modified regions were fabricated with each row corresponding to a specific energy in the range of 40–200 nJ. The row spacing was 3 *μ*m and the spacing between modified regions in a row was 2.5 *μ*m. A 0.55 NA microscope objective (40X magnification) was used to focus light 300 *μ*m below the PMMA surface. Statistical analysis was carried by choosing a row of 20 modified regions with a specific energy of 80 nJ. Similar results were obtained at different pulse energies.

### Thermal degradation

PMMA sample was annealed for an hour at a specific temperature in a Barnstead Thermolyne-1400 Furnace to study thermal degradation of the recorded images. The fluorescence was recorded with the confocal microscope after the annealed sample was cooled to room temperature over several hours. For this study we used an embedded 3-bit image of Albert Einstein fabricated 250 *μ*m below the surface with a 0.9 NA water immersion objective. The laser pulse energy was varied in the range of 25–130 nJ to record the image. From the recorded images, an integrated fluorescence signal was obtained at each temperature and normalized with that of the room temperature. Pearson correlation of the room temperature image with those at other temperatures was obtained using the co-localization tool in Image J. Correlation coefficient of unity corresponds to two identical images while a value of zero corresponds to no overlap between the two images.

### Fluorescence life time Imaging (FLIM)

Images were recorded using a confocal fluorescence microscope (Microtime 200, PicoQuant) system equipped with a frequency doubled picosecond pulse diode laser (485 nm, 100 ps, 40 MHz, LDH-D-C-485, PicoQuant). The FLIM system was used with configuration explained in ref. 36. Microstructures were fabricated near the surface of PMMA at 250 nJ, scan speed of 1 mm/s using 0.25 NA objective. The sample was placed on top of a clean coverslip positioned onto an oil immersion TIR objective (100X, NA 1.45, Olympus, PLAPO) and a beam splitter (500dcxr, Chroma) was used to reflect the excitation light onto the objective after collimation by a fiber optic cable. The epifluorescence signal was passed through a 510 nm long pass filter (Chroma). The fluorescence intensity images and lifetime traces were recorded using a single-photon-counting module (*τ*-SPAD-100, PicoQuant) and Time Correlated Single Photon Counting Module (TSCPC), and processed through SymPhoTime program (PicoQuant).

## Additional Information

**How to cite this article**: Kallepalli, D. L. N. *et al.* Ultra-high density optical data storage in common transparent plastics. *Sci. Rep.* **6**, 26163; doi: 10.1038/srep26163 (2016).

## Supplementary Material

Supplementary Information
