# Supplementary material for: Ultra-high density optical data storage in common transparent plastics
Source: Sci Rep. 2016 May 25;6:26163. doi: 10.1038/srep26163 (PMC4879561; doi:10.1038/srep26163)
Supplement: Supplementary Information [file srep26163-s1.doc]

**Ultra-high density optical data storage in common transparent plastics**

**^1^Deepak L N Kallepalli, ^1,3^Ali M Alshehri, ^2^Daniela T. Marquez, ^1^Lukasz Andrzejewski, ^2,*^Juan C. Scaiano, ^1,*^Ravi Bhardwaj**

^1^Department of Physics, Advanced Research Complex, 25 Templeton Street, University of Ottawa, Ottawa, K1N6N5, Ontario, Canada

^2^Department of Chemistry and Centre for Catalysis Research and Innovation, University of Ottawa, 10 Marie Curie, Ottawa, K1N 6N5, Ontario, Canada

^3^Department of Physics, King Khalid University, P.O. Box 9004, Abha, Saudi Arabia

^*^E-mail: [titoscaiano@me.com](mailto:titoscaiano@me.com), [ravi.bhardwaj@uottawa.ca](mailto:ravi.bhardwaj@uottawa.ca); Phone: +1-6135625800 Ext.6759

**1. 5-bit encoding dynamics at 405 nm excitation.** Blu-ray technology utilizes 400 nm light to read the data on a disc instead of 488 nm that was used in our experiments. To demonstrate the adaptability of our technique with the existing technology we studied the feasibility of multi-level encoding with 405 nm excitation of the read laser. Supplementary Figure 1a shows the fluorescence emitted by these modified regions in the window 425 – 475 nm. The fluorescence signal was recorded and analyzed over 15 – 20 modified regions for each specific energy using Image J. Supplementary Figure 1b shows the evolution of fluorescence signal with the pulse energy of the write laser. The linear variation of the fluorescence signal enables division into different grey levels shown along the right ordinate of Supplementary Fig. 1b. The behavior of fluorescence signal at 405 nm laser excitation is similar to that obtained at 488 nm write laser. Both excitations enabled us to assign 32 levels of grey corresponding to 5 bits of data.


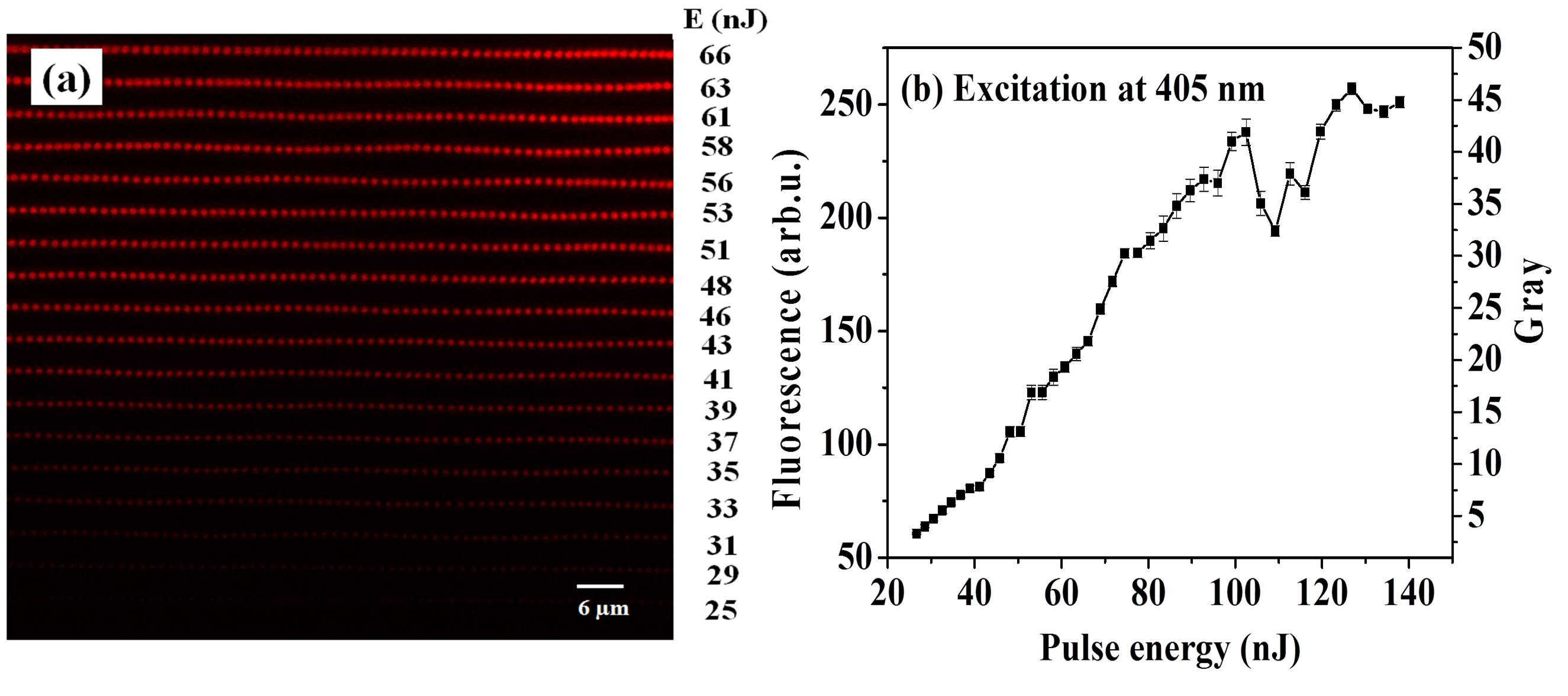


**Supplementary Figure 1.** **5-bit encoding dynamics. a.** Confocal fluorescence microscope image of ultra-fast laser modified regions fabricated using 60X (0.9 NA) water immersion microscope objective and recorded at 405 nm wavelength (emission window 425- 475 nm) with 25X (NA 1.1, working distance 2 mm) Nikon water immersion microscope objective. Energy of each series of modifications is shown on right panel. **b.** Plot of evolution of fluorescence signal with pulse energy. Grey level assigned to fluorescence is shown on the right ordinate.

**2. Generic feature of fluorescence in ultrafast laser treated plastics.** Studies were extended to other transparent plastics to test the generic and ubiquitous feature of laser induced photosensitization in plastics. Series of fluorescent dots were fabricated with 0.55 NA (40X) microscope objective in 5 mm thick (area of 10 mm x 20 mm) Polycarbonate sheet (PC - Goodfellow UK) and Polystyrene (PS- Fisherbrand petridish) in the energy range of 50 – 520 nJ, 100 µm below the surface with 2 µm pitch between two successive dots. Each series was fabricated at a specific energy and two successive series of dots were separated by 5 µm. These fluorescent dots were read using confocal fluorescence microscope at 488 nm (500 – 550 nm emission window; sensitivity of 157 V and 100 µW of excitation power) and 405 nm light (425 – 475 nm emission window; sensitivity of 100V and 328 µW of excitation power). Fluorescence signal was recorded from multiple optical sections of the modified region and stacked for analysis using Image J by averaging over 10 – 15 dots for each laser energy at the excitation of 488 nm.

Supplementary Figures 2a, and 2b show the evolution of fluorescence signal with laser energy in PC and PS at 488 nm laser excitation, respectively. The insets show their chemical structures. Both show linear variation of the fluorescence signal with write laser pulse energy. However, the fluorescence signal in modified PS was small compared with PC. As a result the possible number of grey levels would be small in PS compared to PC or PMMA. Both PC and PS contain aromatic (benzene) rings. We believe that upon ultrafast laser irradiation these polymer chains break up and form loose aromatic double bond structures that give rise to fluorescence emission. Earlier research11 on UV exposure of PS showed formation of three possible double bonded aromatic structures namely trans-stilbene, diphenylbutadiene (DPBD), and diphenylhexatriene (DPHT), all of them exhibited fluorescence emission in the 330 – 520 nm range with absorption bands in 280 – 460 nm. It is therefore possible that multi-photon excitation at 800 nm wavelength in our case can cause similar dynamics. Fluorescence from irradiated plastics is not unique to ultrafast lasers. It has been observed when plastics were exposed to UV, gamma and electron beams^1-7^. However, the unique capability of ultrafast lasers to localize the emission to micron volumes enables high-density data storage.

**
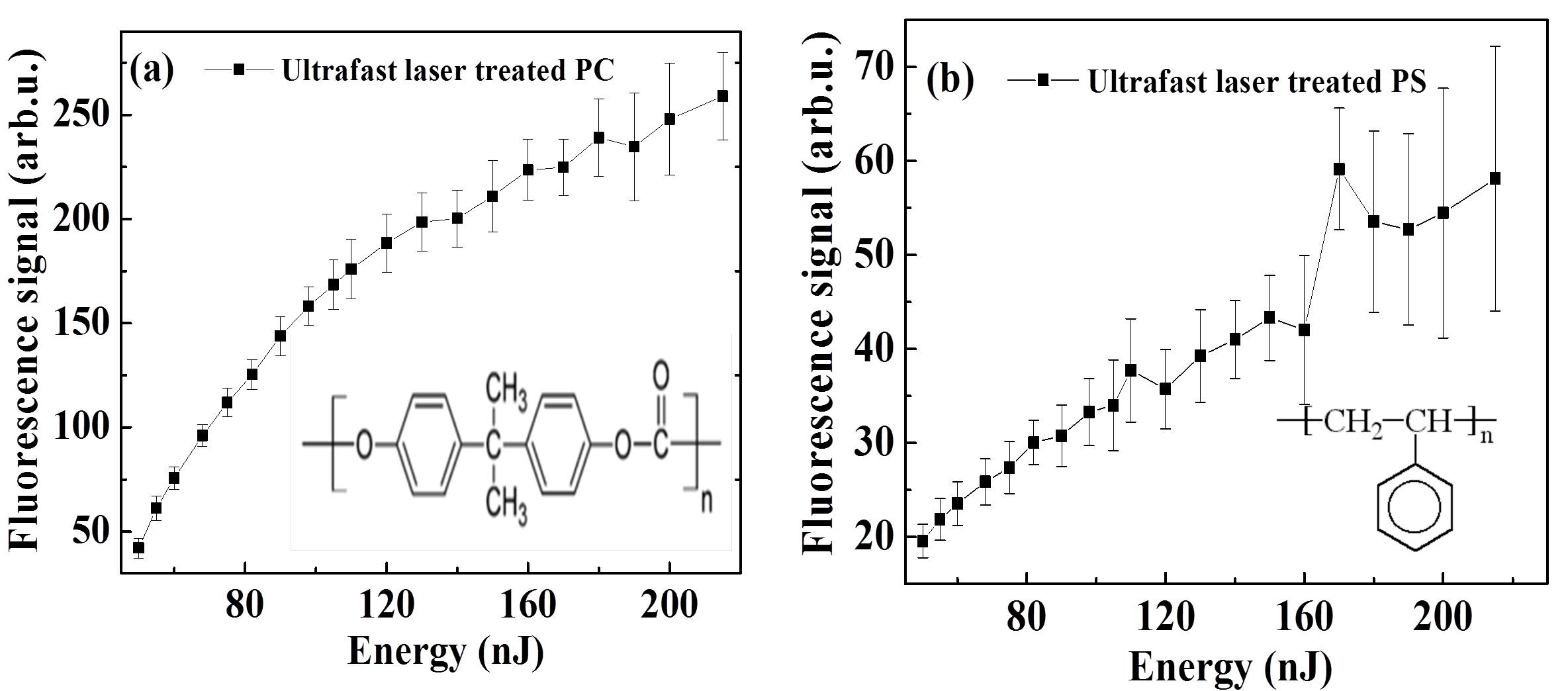
**

**Supplementary Figure 2.Generic feature of fs-laser induced Fluorescence in all plastics.** Evolution of Fluorescence signal with the pulse energy of ultrafast laser in PC **(a)** and PS **(b).** Chemical structures are shown in inset. The laser-modified regions were excited with 488 nm light.

**3. Packing density** is governed by the pixel and layer spacing. The optimal spacing is determined by the requirement of minimal cross-talk between adjacent pixels and image layers. Pixels fabricated at different laser pulse energies varied in size as shown in Supplementary Fig. 3. The pixel sizes were determined by imaging the fluorescence using a confocal microscope. A bit spacing of 1.4 µm used in the experiments was determined primarily by the maximum pulse energy at which (a) the fluorescence signal variation was still linear (as in Figure 1c), and (b) two adjacent bits did not overlap. We used maximum pulse energy of 130 nJ corresponding to a bit size of 1.2 µm. Therefore, a spacing of 1.4 µm ensures no physical overlap between two bits representing the highest grey level.

**Supplementary Figure 3. Pixel size.** Variation of pixel size with laser pulse energy.

To estimate the maximum feasible package density and hence the disc storage capacity, we fabricated embedded images (300 µm below surface) of 32-grey of Albert Einstein, Richard Feynman, and maple leaf with a 10 µm separation in PMMA using 0.9 NA (60X) water immersion microscope objective as shown in Supplementary Fig. 4. The images were downsized to reduce fabrication time. We observed a slight signal overlap between adjacent layers from lateral cross-section of the stack shown in bottom-right of Supplementary Fig. 4.


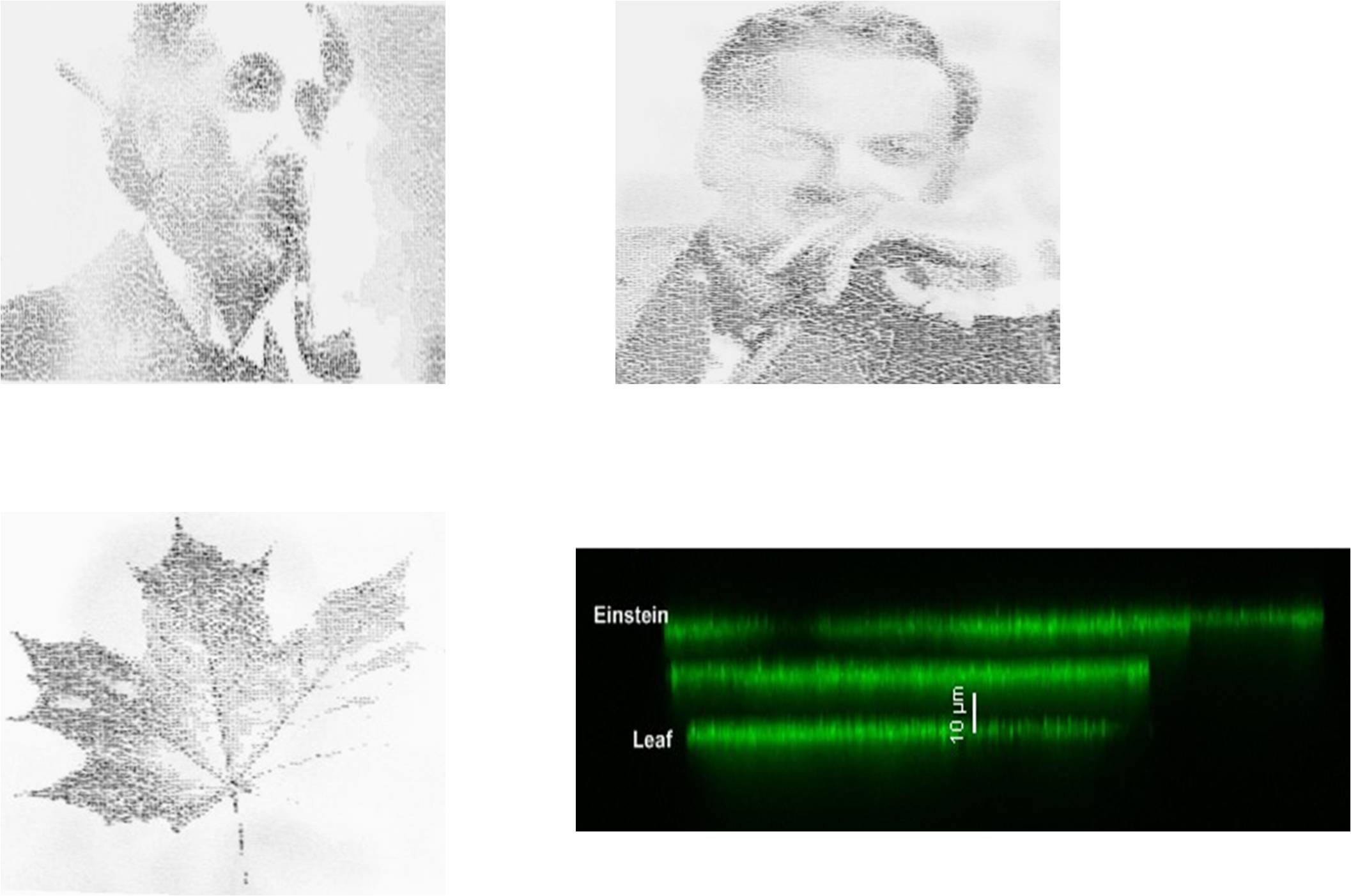


**Supplementary Figure 4. Package density.** 32-grey level embedded images of Albert Einstein (145 pixels x 87 pixels) and Richard Feynman (87pixels x 122 pixels) (shown in top row), and maple leaf (115 pixels x 110 pixels) (shown in bottom left). These images were fabricated with 15 µm separation as shown in lateral cross-section image of the stack in bottom left. Images were obtained from single intense planes of the optically sectioned stacks. This figure is not covered by the CC BY license [Credits to Getty Images for Einstein, Getty Images for the Maple leaf, Mary Evans Picture Library/The Canadian Press for Richard Feynman]. All rights reserved, used with permission.

**Supplementary References**

1. Alshehri, A. M. et al. Localized nanoclusters formation in PDMS upon the irradiation with femtosecond laser. *Opt. Mater. Express.* **5**, 858 – 869 (2015).
2. Demchenko, A. P. The red-edge effects: 30 years of exploration. *Luminescence.* **17**, 1942 (2002).
3. Nie, Z. et al. Multilayered optical bit memory with a high signal-to-noise ratio in fluorescent polymethylmethacrylate. *Appl. Phys. Lett****.* 94**, 111912 (2009).
4. Dickens, B., Martin, J.W. &Waksman, D. Thermal and photolytic degradation of plates of poly (methyl methacrylate) containing monomer. *Polymer.* **25**, 706 – 715 (1984).
5. Nurmukhametov, R. N., Volkova, L. V. & S. P. Kabanov, Fluorescence and Absorption of Polystyrene exposed to UV laser radiation. *J. Appl. Spectrosc.* **73**, 55 – 60 (2006).
6. Deepak, K. L. N., Kuladeep, R., Venugopal Rao, S. & Narayana Rao, D. Luminescent microstructures in bulk and thin films of PMMA, PDMS, PVA, and PS fabricated using femtosecond direct writing technique*. Chem. Phys. Lett.* **503**, 57 – 60 (2011).
7. Barrios, C. A. Fabrication of luminescent nanostructures by electron-beam direct writing of PMMA

resist*. Mater. Lett.* **88**, 93 – 96 (2012).
